# Effect of intrafraction adaptation on PTV margins for MRI guided online adaptive radiotherapy for rectal cancer

**DOI:** 10.1186/s13014-022-02079-2

**Published:** 2022-06-21

**Authors:** Chavelli M. Kensen, Tomas M. Janssen, Anja Betgen, Lisa Wiersema, Femke P. Peters, Peter Remeijer, Corrie A. M. Marijnen, Uulke A. van der Heide

**Affiliations:** Department of Radiation Oncology, The Netherlands Cancer Institute, Plesmanlaan 121, 1066 CX Amsterdam, The Netherlands

## Abstract

**Purpose:**

To determine PTV margins for intrafraction motion in MRI-guided online adaptive radiotherapy for rectal cancer and the potential benefit of performing a 2nd adaptation prior to irradiation.

**Methods:**

Thirty patients with rectal cancer received radiotherapy on a 1.5 T MR-Linac. On T2-weighted images for adaptation (MRI_adapt_), verification prior to (MRI_ver_) and after irradiation (MRI_post_) of 5 treatment fractions per patient, the primary tumor GTV (GTV_prim_) and mesorectum CTV (CTV_meso_) were delineated. The structures on MRI_adapt_ were expanded to corresponding PTVs. We determined the required expansion margins such that on average over 5 fractions, 98% of CTV_meso_ and 95% of GTV_prim_ on MRI_post_ was covered in 90% of the patients. Furthermore, we studied the benefit of an additional adaptation, just prior to irradiation, by evaluating the coverage between the structures on MRI_ver_ and MRI_post._ A threshold to assess the need for a secondary adaptation was determined by considering the overlap between MRI_adapt_ and MRI_ver._

**Results:**

PTV margins for intrafraction motion without 2nd adaptation were 6.4 mm in the anterior direction and 4.0 mm in all other directions for CTV_meso_ and 5.0 mm isotropically for GTV_prim_. A 2nd adaptation, applied for all fractions where the motion between MRI_adapt_ and MRI_ver_ exceeded 1 mm (36% of the fractions) would result in a reduction of the PTV_meso_ margin to 3.2 mm/2.0 mm. For PTV_prim_ a margin reduction to 3.5 mm is feasible when a 2nd adaptation is performed in fractions where the motion exceeded 4 mm (17% of the fractions).

**Conclusion:**

We studied the potential benefit of intrafraction motion monitoring and a 2nd adaptation to reduce PTV margins in online adaptive MRIgRT in rectal cancer. Performing 2nd adaptations immediately after online replanning when motion exceeded 1 mm and 4 mm for CTV_meso_ and GTV_prim_ respectively, could result in a 30–50% margin reduction with limited reduction of dose to the bowel.

## Introduction

Neo-adjuvant (chemo)radiotherapy plays an important role in the multidisciplinary treatment of rectal cancer [1], primarily aiming to reduce local recurrence rates [2, 3] and to downstage the tumor prior to surgery. Accurate radiotherapy delivery to the tumor and elective lymph nodes is hampered by geometrical uncertainties arising from delineation uncertainty, and inter- and intrafraction anatomical variations. To accommodate these uncertainties, the clinical target volume (CTV) is expanded to a planning target volume (PTV). This target volume typically overlaps with the organs at risk (OAR) such as the bladder and the small bowel, resulting in high OAR dose and consequent toxicity [4, 5]. Studying mesorectum motion is important for optimizing rectal cancer radiotherapy in which the mesorectum receives a homogeneous dose in either short or long treatment schedules. Within the context of organ preservation for intermediate and high risk rectal cancer patients, safe dose escalation to the primary tumor may be enabled with the use of smaller PTV margins around the GTV [6].

Recently, integrated MRI linear accelerators were introduced, allowing the use of MRI for online image guidance. With MRI-guided radiotherapy (MRIgRT) high soft tissue contrast images can be acquired at several time points during the treatment which enables daily online adaptation to anatomical changes between treatment fractions and monitoring of anatomical changes during treatment [7]. By planning using delineations of the anatomy on images acquired just prior to the treatment, MRIgRT allows for reduction of geometrical uncertainties due to interfraction motion. As a result of daily online adaptation, intrafraction motion and delineation uncertainty are the primary remaining uncertainties [8]. Online adaptation for rectal cancer is time-consuming with a median duration of 36 min [9] as it requires online redelineation and plan optimization based on the image of the day. As demonstrated by Kleijnen et al. [10], intrafraction motion increases with time requiring larger PTV margins for longer treatment durations. Ideally, to reduce intrafraction motion, online adaptation could be accelerated by automated methods, like auto-contouring or auto-planning, however these methods are still in development for routine clinical use [11].

Strategies for intrafraction motion monitoring and subsequent motion management, including beam gating and multi-leaf collimator tracking, allow for the reduction of uncertainties arising from intrafraction motion. With gating, the target position is monitored continuously and radiation is only delivered if the target is within a pre-defined envelope. Gating has been widely implemented, but its application is mostly limited to periodic motion [12, 13]. Although rectal motion is non-periodic, gating has been applied for mitigating rectal intrafraction motion [14]. Next to gating, tracking has been investigated [15, 16], although it is not clinically available to date on MRI linear accelerators. An alternative, simpler, intrafraction adaptation strategy is to acquire a verification MRI to evaluate target motion during redelineation and plan adaptation, and perform a 2nd adaptation if the target has moved outside a pre-defined envelope. This adaptation can be done by repeating the workflow for the initial adaptation. Adapting based on the verification MRI will probably provide a better surrogate for the anatomy on the post treatment MRI than the adaptation MRI considering the shorter time interval between the scans. As a result of the shorter time interval, target motion may possibly be smaller and a further reduction of PTV margins may be possible. Margin reduction has been studied for prostate [17, 18], lung [19], cervical [20, 21] and spine irradiation [22], however no studies focusing on the potential benefit of intrafraction motion management on PTV margins for rectal cancer were found.

The aim of this work was therefore to determine the PTV margins required to accommodate intrafraction motion of the mesorectum during standard MRIgRT and of the primary tumor during dose-escalated MRIgRT of rectal cancer and secondly to determine the potential benefit of performing a 2nd adaptation prior to irradiation.

## Material and methods

### Patient data

**Fig. 1:**
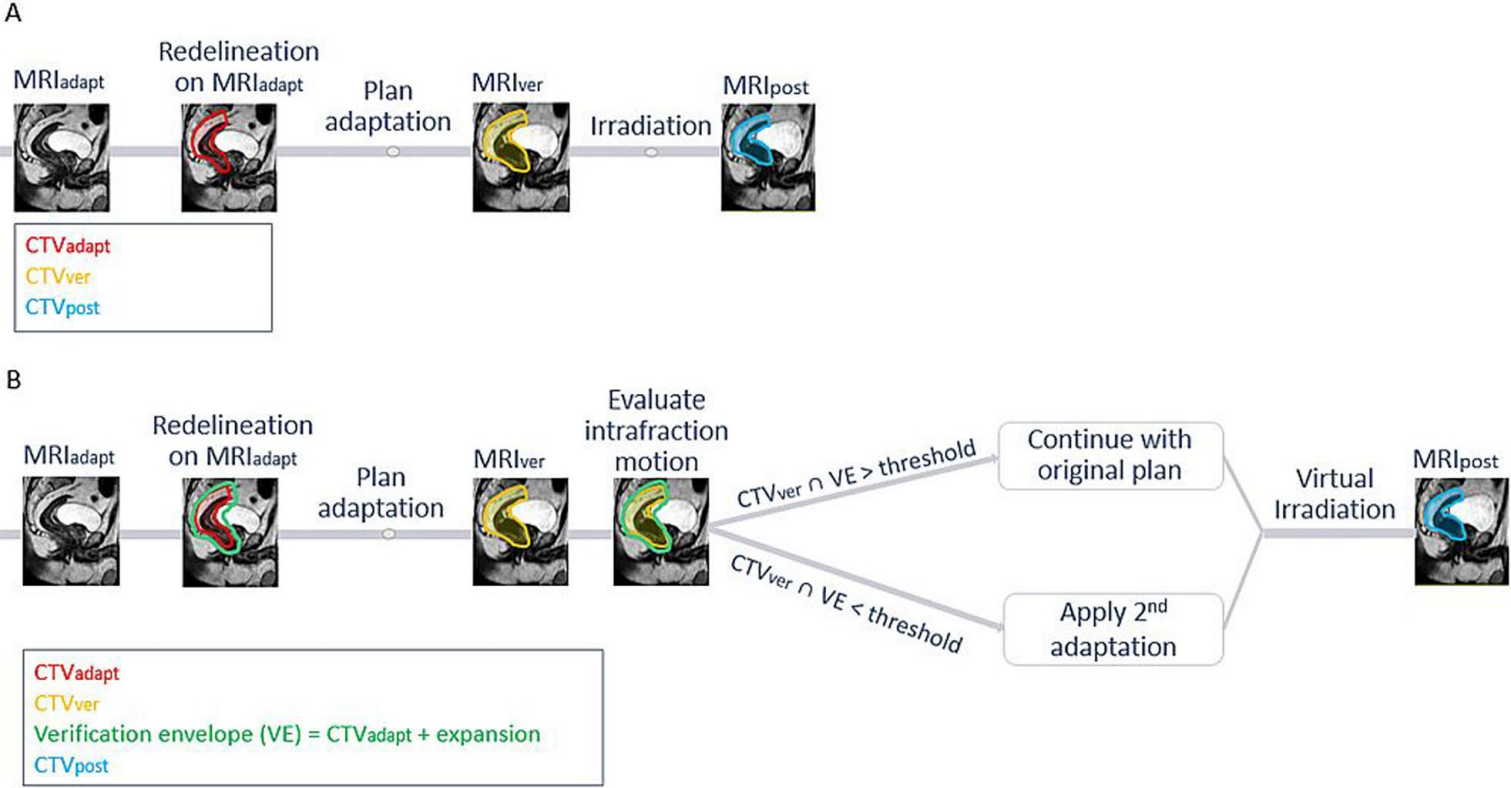
Online adaptive workflow on the MR-Linac A. without 2nd adaptation and B. with motion monitoring and 2nd adaptation after verification. CTV_adapt_, CTV_ver_ and CTV_post_ correspond to the CTV_meso_ on the different scans acquired during the treatment. Steps not relevant to current study are not included in the workflow

Data of 30 patients with intermediate risk or locally advanced rectal cancer treated on a 1.5 T MR-Linac (Unity, Elekta AB, Stockholm Sweden) between October 2018 and March 2021 were analyzed. Twenty-two patients received short course radiotherapy (SCRT; 5 × 5 Gy) and 8 received long course chemoradiotherapy (LCRT; 25 × 2 Gy). Ethics approval was obtained and all patients provided written informed consent for use of their data. Patients were treated using an online adaptive workflow (Fig. 1A) [23]. Approximately a week before start of the treatment, a planning CT and MRI were acquired on which the elective target volumes and organs at risk (OAR) were delineated for treatment planning according to delineation guidelines [24, 25]. For SCRT patients the CTV consisted of the mesorectum, the presacral region and pelvic lymph node regions, while for LCRT patients the lymph node region included the obturator region if pathological lymph nodes in situ were identified. The PTV was generated by adding an anterior anisotropic margin for the mesorectum CTV and a uniform 5 mm margin for the lymph node regions as suggested by Valentini et al. [24]. A bladder filling protocol consisting of drinking 250 ml water 30 min prior to simulation and the radiotherapy session on the MR-Linac was advised. On the MR-Linac, during each fraction first a 3D-T2-weighted MRI was acquired for adaptation (MRI_adapt_) and the planning CT was deformably registered to MRI_adapt_ after which the elective target volumes were re-delineated for SCRT patients. Based on these new delineations, a new plan was optimized. For LCRT the plan corresponding to the actual mesorectum shape was selected through the use of a library of plans [26], followed by a virtual couch shift to correct for set-up errors[23]. Prior to starting the irradiation, an additional MRI was acquired to verify the position of the structures (MRI_ver_). Subsequently, all patients were irradiated with an optimized 9-field intensity modulated radiotherapy (IMRT) plan with beam avoidance angles for the cryostat pipe (gantry angles 8°-18°) and two high attenuation regions of the MRL treatment couch (100°–140° and 220°–260°) [27]. After irradiation, a post treatment MRI (MRI_post_) was acquired.

All acquisitions were performed with Field of View (FOV): 400 × 448 × 249 mm^3^, repetition time (TR): 1300 ms, echo time (TE): 128 ms, MRI_adapt_ had voxel size of 1.2 × 1.2 × 1.2 mm^3^ and acquisition time of 6 min, while MRI_ver_ and MRI_post_ used 1.2 × 1.2 × 2.4 mm^3^ acquired in 3 min.

All available images acquired during 5 daily treatment fractions of the patients treated in a short course scheme and the first fractions of every week of patients treated in a long course scheme were used for this study. The time intervals between MRI_adapt_ and MRI_ver_, and MRI_ver_ and MRI_post_ were determined. These intervals correspond to the time needed for recontouring and plan adaptation, and irradiation respectively. On all the images, the gross tumor volume of the primary tumor (GTV_prim_) and the mesorectum clinical target volume (CTV_meso_) were delineated retrospectively using the contouring toolbox in Monaco v5.40.01 (Elekta, Stockholm, Sweden) by 2 experienced radiation technology therapists (RTT) following delineation guidelines [24, 25]. For each fraction, delineations of MRI_adapt_ were copied to the MRI_ver_ and MRI_post_, and manually adjusted. All scans of one patient were delineated by the same RTT. Delineations were verified and, if needed, corrected by a radiation oncologist with over 10 years’ experience. We indicate the mesorectum CTV as delineated on the MRI_adapt_ with CTV_meso,adapt_ in the remainder of this paper. We use a similar convention for the other scans.

The peritoneal cavity (bowel area) as delineated on MRI_adapt_ of the first fraction was used and adjusted if needed. The CTV of the elective lymph node regions was not included in this study, considering the intrafraction motion of these regions is expected to be small [9]. For the same reason, a 2nd adaptation is suspected to have no substantial effect.

### PTV margin determination

For every fraction, the delineated structures (GTV_prim,adapt_ and CTV_meso,adapt_) were expanded in 3D to new structures: PTV_prim_ and PTV_meso_ in steps of 1.0 mm. The expansions were obtained using a rolling-ball algorithm [28], where the expansions were simulated on the actual scan. For GTV_prim,adapt_ the margin was isotropic and for CTV_meso,adapt_ we used anisotropic margins with the anterior expansion 1.6 times larger compared to all other directions. This choice is motivated by the study of Nijkamp et al. on mesorectum shape variation [29]. For every expansion the coverage was determined between the PTV and the associated structure on MRI_post_. CTV_meso_ and GTV_prim_ were analyzed separately and independently. The coverage was defined as the number of overlapping voxels of the PTV and corresponding structures on MRI_post_ as a percentage of the total number of voxels of the structure on MRI_post_. For CTV_meso_, we considered the margin adequate when on average over the 5 fractions, PTV_meso_ covered 98% of CTV_meso,post_ in 90% of the patients. For the boost to the GTV_prim_ the criterion was relaxed to 95% volumetric coverage of GTV_prim,post_ by PTV_prim_ in 90% of the patients. The reason for using a lower coverage criterion is because the GTV_prim_ would be irradiated as a boost on top of the irradiation of the CTV_meso_, resulting in less steep dose gradients. Considering the heuristic choice of these coverage criteria, we also assessed the effect of different volumetric coverage criteria on the PTV margins. All expansion and coverages were calculated using in-house software Match42.

### Second adaptation

To study the effect of a 2nd adaptation after MRI_ver_, the intrafraction motion during redelineation and plan adaptation was evaluated. The verification envelope (VE) is defined as an expansion around GTV_prim,adapt_ or CTV_meso,adapt_. We consider the threshold for intrafraction motion during adaptation to be exceeded, when the VE does not cover the GTV_prim,ver_ or CTV_meso,ver_ for at least 95% and 98% respectively.

For an isotropic VE, we determined whether the intrafraction motion during redelineation and plan adaptation exceeded the threshold for GTV_prim_. For CTV_meso_ the VE was anisotropic with a similar anterior expansion a factor 1.6 times all other directions. If the threshold was not exceeded, no 2nd adaptation would be needed and thus GTV_prim,adapt_ or CTV_meso,adapt_ was used for evaluation. If the threshold was exceeded, a 2nd adaptation was applied and GTV_prim,ver_ and CTV_meso,ver_ were used to generate a new PTV_prim_ and PTV_meso_ to represent this adaptation. The online adaptive workflow following this approach is shown in Fig. 1B.

**Fig. 2:**
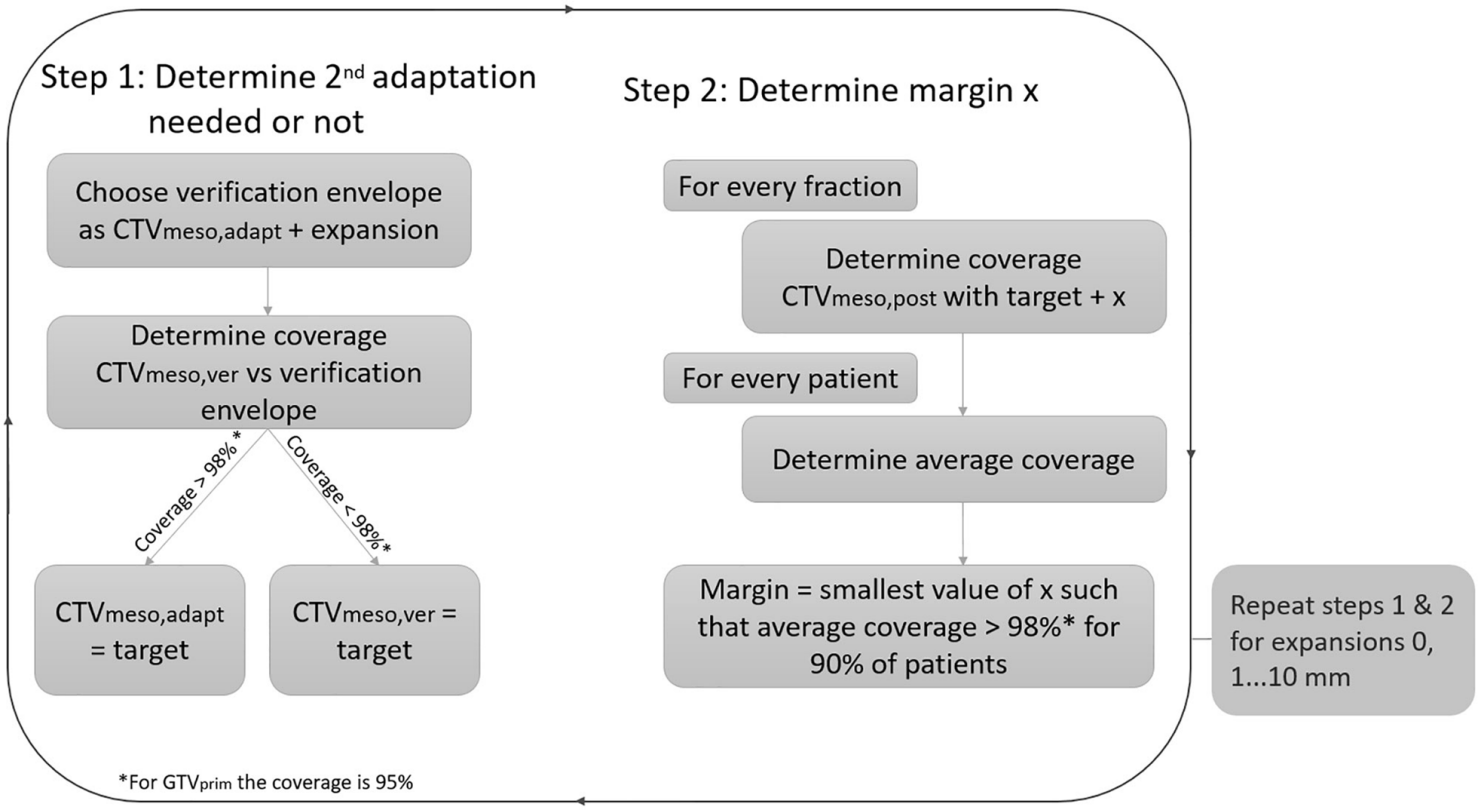
Method used to determine percentage of 2nd adaptations and the resulting margin. Different expansions for the envelope are chosen. For each value we first determine whether to perform a 2nd adaptation and next we determine the required margin

We varied the VE between 0 and 10 mm with 1 mm increments and we determined for each size the required PTV margin and the frequency of 2nd adaptations needed. This process is summarized in Fig. 2.

To determine the potential benefit of a 2nd adaptation for organs at risk, we determined the overlap between the bowel area and the required PTV_meso_ when no 2nd adaptations are performed and compared this to the overlap between the bowel area and PTV_meso_ after 2nd adaptations.

As a surrogate for the time required for re-delineation during the first and 2nd adaptation, we consider the change in volume of the CTV_meso._ This is a practical surrogate, since re-delineation of CTV_meso_ is in particular necessary due to changes in rectal filling, which can directly influence the volume. For the first adaptation we consider the volume change between the reference CT and MRI_adapt_. For the 2nd adaptation we consider the change between MRI_adapt_ and MRI_ver._ A paired sample t-test was carried out (in SPSS v27.0) to test for a significant difference (α = 0.05).

## Results

For 4 out of 30 patients one or more MRI _post_ were not available and one patient received a treatment fraction on the conventional linear accelerator resulting in a total of 144 fractions available for analysis. For one patient the GTV_prim_ was poorly visible on MRI and therefore not delineated, resulting in a total of 139 fractions for analysis of GTV_prim_. Patient and tumor characteristics are summarized in Table 1.

**Table 1:** Baseline patient and tumor characteristics

Patient characteristics	N = 30 (%)
**Age in years (median; range)**	61; 34–77
**Sex**	
Male	20 (66.7)
Female	10 (33.3)
**Tumor stage**	
cT2	9 (30.0)
cT3	20 (66.7)
cT4	1 (3.3)
**Nodal stage**	
cN0	15 (50.0)
cN1	11 (36.7)
cN2	4 (13.3)
**Tumor location (distance to anorectal junction)**	
Lower rectum (0 to ≤ 5 cm)	22 (73.3)
Mid rectum (> 5 to 10 cm)	6 (20.0)
Upper rectum (> 10 cm)	2 (6.7)
**Evaluable fractions (N = 150)**	
CTV_meso_	144 (96.0)
GTV_prim_	139 (92.7)

Data are displayed as numbers (%) unless indicated otherwise.

### PTV margins and 2nd adaptations

**Fig. 3:**
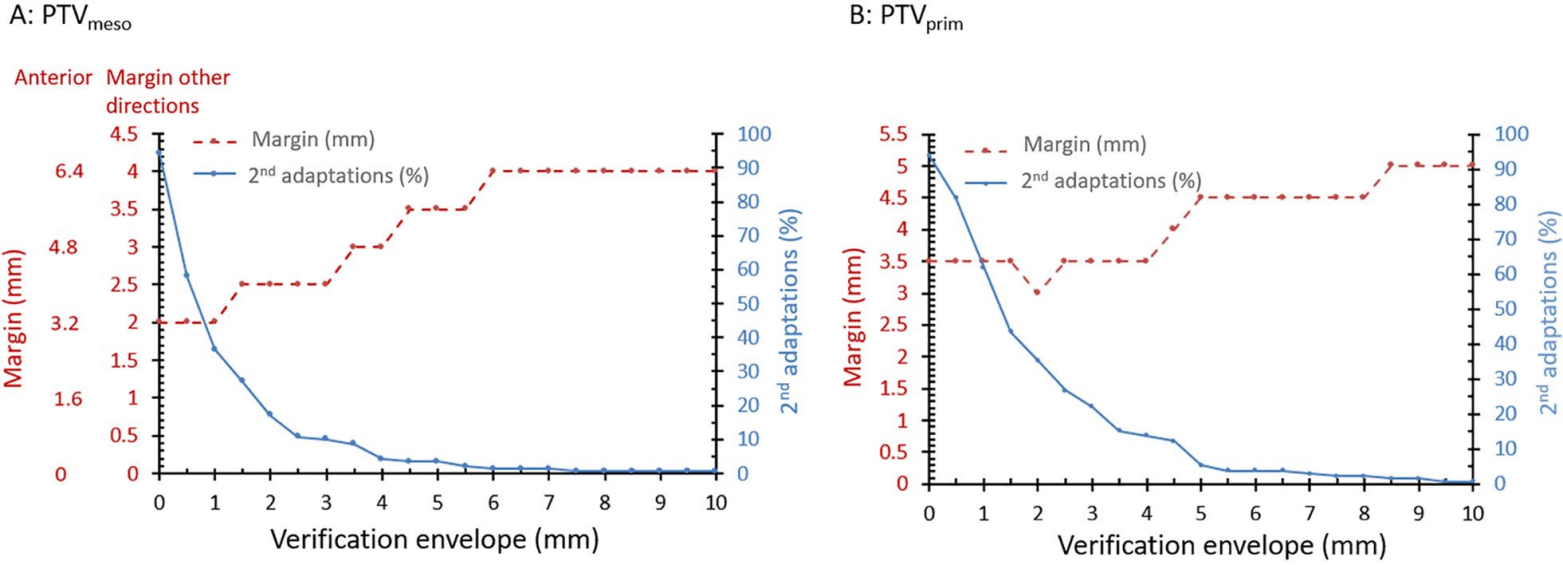
Margins (red line) and percentage of 2nd adaptations (blue line) needed for varying verification envelopes to realize an average coverage of A. 98% for CTV_meso_ & B. 95% for GTV_prim_

The median time between MRI_adapt_ and MRI_ver_ was 12 min (inter-quartile range IQR = 10–23 min) and between MRI_ver_ and MRI_post_ 12 min (IQR = 11–15 min). The margin required for PTV_meso_ without 2nd adaptations was 6.4 mm in the anterior direction and 4.0 mm in all other directions. This is indicated in Fig. 3 for a VE of 10 mm, in which case no 2nd adaptations are needed. For PTV_prim_ a margin of 5.0 mm was required.

**Fig. 4:**
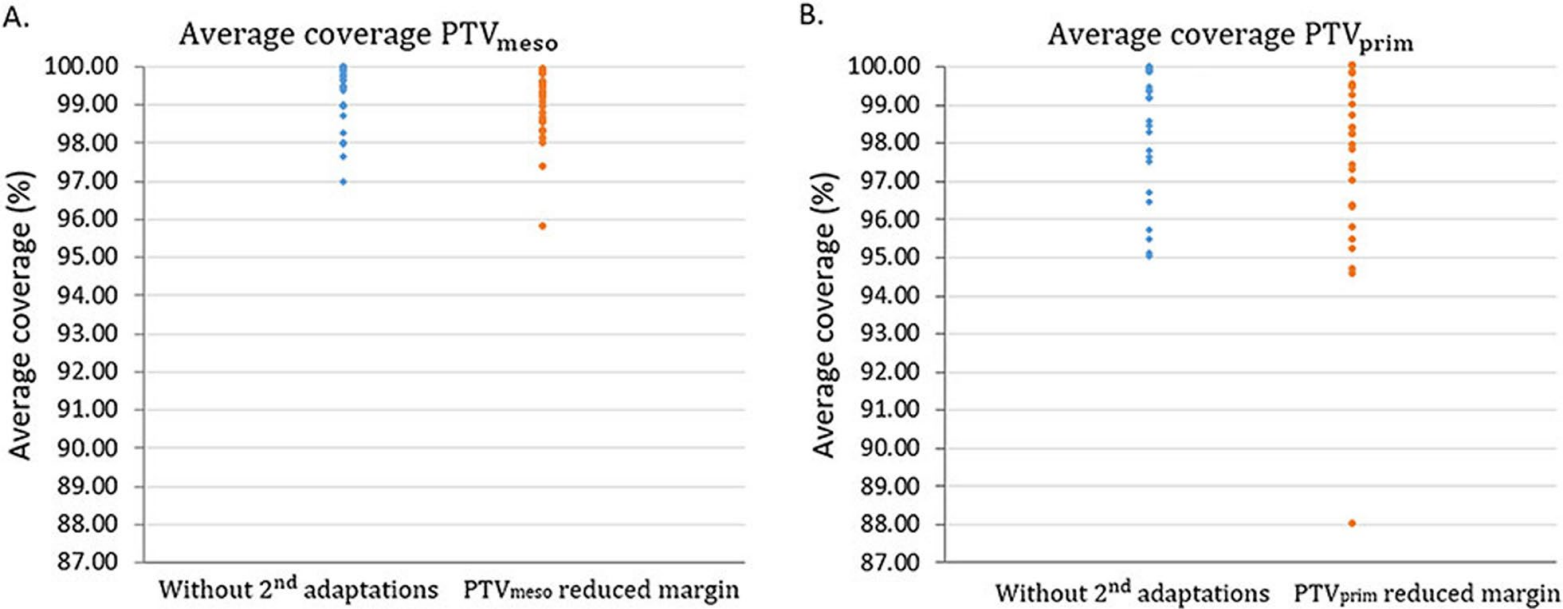
Average coverage for the complete population for A. PTV_meso_ and B. PTV_prim_

The coverage for all patients (n = 30) is shown in Fig. 4. In 90% of the population, the target criteria of 98% and 95% coverage were reached. The coverage of the remaining 10% was somewhat lower, but still above 88% in all cases.

The percentage of 2nd adaptations and resulting PTV margins are shown in Fig. 3. The minimal feasible margin for PTV_meso_ when performing 2nd adaptations was 3.2 mm in the anterior direction and 2.0 mm in all other directions using a VE of 1.0 mm. To achieve this margin, 2nd adaptations needed to be performed in 36% of the fractions.. Adapting more fractions did not lead to further margin reduction. For GTV_prim_, 2nd adaptations would be needed in 17% of the fractions at a VE of 4.0 mm to reduce PTV_prim_ to 3.5 mm. Table 2 shows the number of patients needing a 2nd adaptation in 0, 1,2,3 of 4 fractions. For CTV_meso_ 7 patients needed a 2nd adaptation in 3 or more fractions. One of these patients was treated with LCRT. For GTV_prim_, only 2 patients with tumors located in the mid- and upper rectum respectively, required a 2nd adaptation in 3 or more fractions.

**Table 2:** Frequency of 2nd adaptations on a patient-level for the minimum feasible margins

Fractions needing a 2nd adaptation	Number of patients	
	CTV_meso_	GTV_prim_
0	9	15
1	6	11
2	8	1
3	2	2
4	5	-

When no 2nd adaptations were performed, the median (IQR) volume of the bowel that overlapped with PTV_meso_ was 35.3 (25.2–52.4) cm^3^ as shown in Table 3. For the reduced PTV_meso_ that was possible after 2nd adaptations, 19.3 (12.6–34.7) cm^3^ of the bowel overlapped with PTV_meso_.

**Table 3:** Overlapping volume of the bowel with PTV_meso_ without and after 2nd adaptations

	Median overlapping volume (IQR)
Overlapping bowel with PTV_meso_ without 2nd adaptations	35.3 (25.2–52.4) cm^3^
Overlapping bowel with minimum PTV_meso_ after 2nd adaptations	19.3(12.6–34.7) cm^3^

The CTV_meso_ showed larger changes between the planning CT and MRI_adapt_ than between MRI_adapt_ and MRI_ver_, as reflected by a median volume difference (IQR) of 26.1 (14.6–45.7) vs 7.2 (4.8–12.9) cm^3^; p < 0.05.

### Effect of coverage criteria on PTV margins

**Fig. 5:**
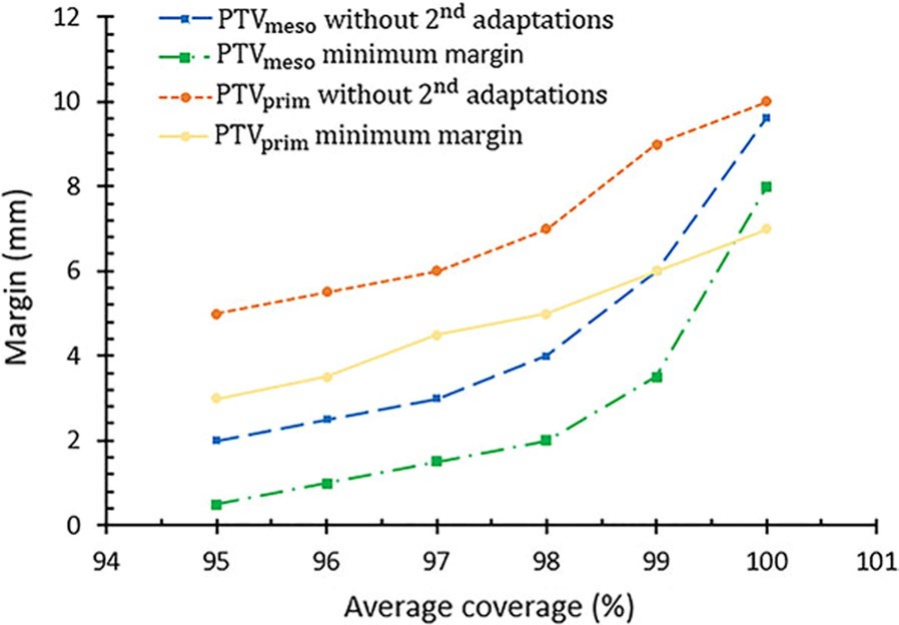
PTV margins without 2nd adaptations and minimum feasible PTV margins when performing 2nd adaptations for different coverage criteria. The anterior margin for PTV_meso_, is a factor 1.6 times the margin in all other directions

For different coverage criteria, PTV margins for a workflow without 2nd adaptations (blue line) and when the minimum feasible margin is reached when performing 2nd adaptations (red line) are shown in Fig. 5. For both scenarios the margin increases gradually as more volumetric coverage is required. Above 97% a steeper increase of the margins is seen for PTV_meso_ as compared to the lower coverage criteria.

## Discussion

The aim of this study was to determine the PTV margins required to accommodate intrafraction motion of the mesorectum (CTV_meso_) and the gross tumor volume (GTV_prim_) during MRIgRT of rectal cancer and to determine if performing a 2nd adaptation prior to irradiation would potentially be beneficial.

For the CTV_meso_ we found a required margin of 6.4 mm in the anterior direction and 4.0 mm in all other directions without a 2nd adaptation. Introducing 2nd adaptations allowed a reduction to 3.2 mm in the anterior direction and 2.0 mm in all other directions. For the GTV_prim_, a PTV margin of 5.0 mm was needed, whereas 2nd adaptations allowed for a reduction to 3.5 mm.

Several studies have reported on the motion of the CTV_meso_ [10, 29–32] and GTV_prim_ [10, 33, 34].

Kleijnen et al. studied the motion uncertainty as a function of time of CTV_meso_ and GTV_prim_ using repeated cine-MRI data of 16 patients [10]. They found PTV margins of 12 mm for intrafraction motion up to 18 min which were comparable in magnitude to margins found for interfraction motion [10]. The differences are likely due to the use of different coverage criteria. In the study of Kleijnen et al., the distance that incorporates 95% of the surface voxels at the investigated time point was required to fit within the margin in 90% of all fractions. In our work the margin was selected for an average volumetric coverage of 95% in 90% of all patients.

With regards to PTV_prim,_ our findings are in line with previous studies [33, 34]. Van de Ende et al. studied the inter- and intrafraction displacement of the GTV based on fiducial markers on cone beam CT images and reported PTV margins of 3.0 mm in left–right direction, 4.7 mm in anterior–posterior direction and 5.5 mm in cranial-caudal direction for intrafraction displacement [33]. In addition, they showed larger motion for proximal tumors as compared to distal tumors and hypothesized that the reduction of required margins may be higher in patients with a proximal compared to a distal tumor.

More recently, Eijkelenkamp et al. determined margins to compensate for intrafraction GTV_prim_ motion during online adaptive procedures [34]. They used a similar method as the current study to determine the required margin for online adaptive MR-guided dose escalation for intermediate risk rectal cancer patients and reported a margin of 6 mm for the entire treatment, which could be reduced to 4 mm for a procedure of 15 min or less. These findings are consistent with the PTV margins found in current study.

Although intrafraction motion for CTV_meso_ and GTV_prim_ has been studied previously, the current study also explores the potential benefit of intrafraction motion management during MRI-guided radiotherapy to reduce the required PTV margins. As shown in the results, adapting just prior to the start of irradiation instead of only at the beginning of the treatment possibly provides a more accurate estimation of the anatomy during irradiation in some cases, given the shorter time interval between MRI_ver_ and MRI_post_ compared to MRI_adapt_ and MRI_post_. For GTV_prim_ relatively more 2nd adaptations were needed to achieve a margin reduction of 30%. This may possibly be attributed to the larger observer variability for the primary tumor as compared to mesorectum [35]. In addition, when considering the number of 2nd adaptations and the resulting margins for different verification envelopes as depicted in Fig. 3, one can make a tradeoff between the workload and the benefit of motion management to reduce the required margins.

When assessing the effect of the margin reduction on bowel toxicity, we showed that the volume of the bowel receiving 95% of the prescribed dose (23 Gy) is reduced with only 16.0 cm^3^ after performing 2nd adaptations. Both before and after 2nd adaptations, the volume of bowel area receiving 23 Gy was lower than the upper limit of 85 cm^3^ recommended by adapted Quantitative Analysis of Normal Tissue Effects in the Clinic (QUANTEC) guidelines [36]. Considering this, the clinical impact of margin reduction as a result of 2nd adaptations might be limited for dose reduction to the bowel. Overall the choice to treat a patient on a MR-Linac systems is carefully considered by clinicians weighing the clinical benefits against the added time and workload. With daily online adaptation and motion management where needed the treatment is tailored to each patients’ anatomy, allowing for more accurate RT, reduced margins and possibly dose escalation.

In this study we introduced a verification envelope (VE) for deciding when to perform a 2nd adaptation. The coverage threshold based on this VE replaces the common practice to take action if the target moves out of the PTV. As demonstrated in the results, the required PTV margin is typically not identical to the VE. Using the PTV margin as envelope can result in performing either too few or too many 2nd adaptations than necessary. The concept of a VE is consistent with motion management techniques such as automated beam gating [37] and target tracking [38] as these are solely based on movement of the target outside of a pre-specified threshold, and do not inherently use the PTV margin for this purpose. Up to date there is one study by Chiloiro et al. [14] demonstrating the clinical feasibility of beam gating in rectal cancer with a region of interest set around the mesorectum.

A limitation of this study was that the duration of the 2nd adaptations was not considered. We assumed an instantaneous adaptation, which is not feasible in clinical practice. When performing the 2nd adaptation, motion may occur during that time period as well. Consequently, the margins found in this study should be considered a lower boundary of what would be achievable. Our method for 2nd adaptations uses new delineations on MRI_ver_, which would be consistent with an Adapt to Shape workflow on the Unity system. Although full redelineation as part of a 2nd adaptation is the most accurate approach to account for intrafractional anatomical changes, this method tends to be time-intensive, but is expected to be faster than the first adaptation. Because we assume the 2nd adaptation to be faster, we used the volume differences between the contours prior to and after adaptation as an estimate for the adaptation time. Here volume differences were used as a surrogate for the added path length [39] and we saw that volume differences were significantly smaller for the 2nd adaptation.

At the time of adapting for the 2nd time, the patient has been on the treatment table for a while and may be more relaxed, possibly resulting in a reduced amount of motion as compared to the first adaptation. An option to limit the adaptation time might be to opt for a less accurate and faster approach such as Adapt to Position [23]. However, a downside is that the Adapt to Position approach only corrects rigid translations of the target volume. Nevertheless, the exact implications of the duration of 2nd adaptation remain to be studied further. Speeding up the first adaptation, specifically delineation, might be the ideal solution. However, automation methods such as auto contouring are still in development.

The criteria for margin determination were based on volumetric coverage and not statistical inferences from accumulated dose as is done in deriving the classical margin recipes [40, 41]. For a comparison with these recipes the local standard deviation of the positioning error should have been determined. However, to translate this into a margin, assumptions on the dose distribution, local distribution of positioning errors and target deformation have to be made. Our volumetric approach is considerably easier to interpret and requires only the choice of a coverage criterion. However to formally asses that, a dose accumulation study needs to be performed. Because of the heuristic nature of these choices we also provided results for different coverage criteria.

The proposed margins primarily account for uncertainties due to intrafraction motion conform the online adaptive workflow. In the total PTV used in clinical practice other uncertainties such as uncertainties in gantry positioning, MLC motion, image alignment should be included. Gantry position and choices related to MLC positioning are typically institute-specific. When using this work to determine margins for clinical practice, care should be taken to ensure that all relevant uncertainties are taken into account.

Given the comparison of two delineations on different scans, the analysis is potentially influenced by delineation variability. However, within a single patient we minimized this variation by having the same observer delineate all scans of one patient. Moreover, for the verification and post treatment scan the delineation was performed by adjusting a copy of the delineation on the adaptation scan, minimizing the delineation variability within a single fraction.

## Conclusion

Our study shows that the PTV margins to accommodate intrafraction motion of the mesorectum in online adaptive MRIgRT for rectal cancer are 6.4 mm in anterior direction and 4.0 mm in other directions, and 5.0 mm for the GTV of the primary tumor. In this study we introduced a verification envelope based on which the decision is made on when to perform a 2nd adaptation. Even in the most optimistic scenario motion management in the form of a 2nd adaptation prior to irradiation, these margins can be reduced to 3.2 mm in anterior direction and 2.0 mm in other directions for the mesorectum and 3.5 mm for the primary tumor, with limited reduction of dose to the bowel.

## Data Availability

The datasets generated and/or analyzed during the current study are not publicly available due to protection of individual patient privacy and the use of an in-house software but are available from the corresponding author on reasonable request.

## Abbreviations

CTV: Clinical target volume
GTV: Gross tumor volume
PTV: Planning target volume
OAR: Organs at risk
MRIgRT: MRI-guided radiotherapy
MRI_adapt_: Adaptation MRI
MRI_ver_: Verification MRI
MRI_post_: Post treatment MRI
GTV_prim_: Gross tumor volume of the primary tumor
CTV_meso_: Mesorectum clinical target volume
RTT: Radiation technology therapists
FOV: Field of view
TR: Repetition time
TE: Echo time
VE: Verification envelope
